# Efficacy of a mixture of simethicone and tyndallized bacillus coagulans in infant colic: a pilot study on behalf of Italian Society of Pediatrics (SIP)

**DOI:** 10.1186/s13052-025-02140-2

**Published:** 2025-11-12

**Authors:** Michele Saviano, Marina Russo, Pietro Buono, Michele La Pietra, Elvira Sorrentino, Annamaria Chianese, Stefano Ementato, Giuseppe Illiano, Gerardo Napolitano, Pasquale Dolce, Annamaria Staiano

**Affiliations:** 1 Primary Care Pediatricians, ASL Napoli 3 Sud, Naples, Italy; 2 Department of Translational Medical Science, Section of Pediatrics, “Federico II” University of Naples, Via S. Pansini, 5, Naples, 80131 Italy; 3 Director UOD Consultancy Activies and Maternal and Child Care- Management Center Is. C3, General Direction for Health Protection and Coordination of the Regional Health System, Naples, 80143 Italy

**Keywords:** Infant colic, Abdominal pain, Tyndallized bacillus coagulans, Simeticone, Functional gastrointestinal disorders

## Abstract

**Background:**

Infant colic is a common functional gastrointestinal disorder characterized by excessive crying with no effective treatment available. We aimed to evaluate the efficacy of a mixture of Tyndallized Bacillus Coagulans and Simethicone in reducing the crying time in colicky infants and improving quality of sleep and infants’ and parents’ quality of life.

**Methods:**

A pilot study was conducted on a group of 41 infants with diagnosis of Infant Colic according to Rome IV criteria. We administered to all the enrolled infants a mixture of Tyndallized bacillus coagulans and Simethicone for 28 days. The primary outcome was the mean infant crying duration at 28th day. The secondary outcome was the improvement in the quality of sleep and infants’ and parents’ quality of life.

**Results:**

Forty-one infants were enrolled, two didn’t complete the study. In 89% of patients, we observed at least a 50% reduction in crying time at 28 days post-intervention. This success rate was significantly higher (*p* < 0.001) compared to a physiological reduction in newborn/infant crying, estimated at 39%. We observed that the mean daily crying time at the end of the treatment was significantly lower (*p* < 0.001). Regarding the sleep duration we found a significantly longer diurnal and nocturnal sleep at 28th day (*p* < 0.001 and *p* < 0.001, respectively). In addition, a significant improvement of mothers' and fathers’ quality of life and severity perception of IC was detected at 28th day (*p* < 0.001 and *p* < 0.001, respectively). No relevant adverse events were observed.

**Conclusions:**

Tyndallized bacillus coagulans and Simethicone seems to be promising in the management of infants with colic. Nevertheless, further studies are needed to confirm this preliminary data.

**Trial registration:**

ClinicalTrial.gov, NCT06458881. Registered 11 June 2024—Retrospectively registered.

## Background

Infantile colic (IC) is a common condition, responsible for 25% of pediatric consultation in the first 3–4 months of life, affecting from 5 to 30% of infants between 2 weeks and 3 months of age [1–4]. Despite the high prevalence, the etiology of IC still remains unclear. Gastrointestinal theories include increased intra-abdominal gas, hyperperistalsis and visceral pain [5]. Regarding psychosocial hypotheses, maternal anxiety, depression and difficult infant temperament have been correlated to IC [5]. During the last decade, the composition of the intestinal microbiome has been addressed as an independent risk factor for IC. Several studies indicate that inadequate lactobacilli colonization in the first few months of life may affect intestinal fatty acid profile, favoring the development of IC [6]. Coliform bacteria have also been found more abundantly in colicky infants and it is speculated that altering the composition of the intestinal microbiota may positively influence the management of affected infants. As a matter of fact, research into the use of probiotics for colic has been rapidly gaining momentum recently, but results are conflicting. Infants with colic are reported to have increased concentration of gas-forming organisms and proteobacteria such as Escherichia coli in their gut. Colonization with certain intestinal microorganisms, such as Bifidobacterium and Lactobacillus species, along with increased intestinal microbial diversity, may protect against infant distress. One clinical trial showed safety and efficacy of Lactobacillus reuteri in prevention of IC [7]. A recent meta-analysis of three small, randomized controlled trials, of breastfed infants with IC reported that Lactobacillus reuteri noticeably reduced crying time at 21 days post supplementation [8]. Recently Chau et a., showed that administration of Lactobacillus reuteri DSM 17938 significantly improved colic symptoms by reducing crying and fussing times in breastfed Canadian infants with colic [9]. In contrast, a double blind, placebo controlled randomized trial on the same probiotic strain Lactobacillus reuteri DSM 17938 showed that it did not benefit a community sample of breastfed infants and formula-fed infants with IC [10]. A recent ESPGHAN (European Pediatric Society of Gastroenterology, Hepatology and Nutrition) position paper on the use of probiotics in Gastrointestinal disorders underlined that healthcare professionals may recommend *L reuteri* DSM 17938 (108 CFU/day for at least 21 days) for the management of infant colic in breastfed infants [11]. However, the safety profile with the use of live probiotics is still a matter of debate. The main risks include: cases of systemic infections due to translocation, particularly in vulnerable patients and pediatric populations; acquisition of antibiotic resistance genes; or interference with gut colonization in neonates. To avoid these risks, there is an increasing interest in non-viable microorganisms or microbial cell extracts to be used as probiotics, mainly heat-killed (including tyndallized) probiotic bacteria (lactic acid bacteria and bifidobacteria). The process of tyndallization consists in a 1-h long heat treatment at 70 °C on three consecutive days and in gamma irradiation of lactobacilli [12]. This procedure guarantees the killing of the live bacteria and the preservation the produced probiotics [13]. Focusing on pharmacological treatments, simethicone, which reduces gas production, may actually be helpful for some infants, although several randomized controlled trials noted no difference in reducing colic episodes compared with placebo [14, 15]. However, current literature does not recommend the use of any drugs because of reported side effects.

The aim of our preliminary study was to evaluate the efficacy of an association of Simethicone and tyindallized Bacillus coagulans in reducing the infant crying duration in IC.

## Methods

This pilot study is a prospective study coordinated by the Department of Translational Medicine, Section of Pediatrics, University of Naples “Federico II”, whereas infants were recruited by 8 general pediatricians belonging to the Pediatrics Investigator Committee of Campania Region, Italy. Inclusion criteria were: full term infants (> _37 weeks gestation at birth; 5-min Apgar score > _7; Birth weight > _2500 g), aged < 12 months, diagnosed with IC according to Rome IV criteria [1], breast- and formulafed infants. The parents of the infants participating in the study were supplied with the mixture of Tyndallized B. Coagulans and Simethicone. The Institutional Review Board of Campania 3 approved the research protocol (Number 91/2023). All parents gave written informed consent. Enrolled infants were treated for 28 days with a mixture of Tyndallized B. Coagulans and Simethicone: 20 drops, four times a day. At enrolment, clinical and dietary history, obstetrical data and anthropometry were recorded. The subjects were classified as having IC based on their parents' responses to the validated questionnaires regarding IC according to Rome IV criteria. The parents were also asked to fill in the following questionnaires: 1) Baby's Day Diary on daily crying and infant sleep duration [16]; 2) a scale (visual analogue scale, VAS, 0e10) for parents' quality of life, a questionnaire on infant's quality of life; 3) a form for stool frequency and consistency; 4) a scale for parental perception of colic severity (VAS 0e10) and 5) a scale for parental perception of sleep quality (VAS 0 e10). Infants were evaluated by a physician for follow-up visits at week 4. During the visits, physical examination was performed and information regarding drugs administration, number and site of infections and eventual adverse medical events were recorded. Moreover, parents had to fill: 3) the questionnaire on infant's quality of life; 4) a review of stool frequency and consistency; 5) the parental perception of colic severity (VAS 0e10) and 6) the parental perception of sleep quality (VAS 0e10). All the authors had access to the study data. Compliance was assessed by evaluating the diary provided by the parents.

### Outcomes

The primary endpoint was the infant crying duration at 28 days. Treatment success was defined as at least 50% reduction in crying time from baseline to day 28 post-intervention. Secondary endpoints included: infant sleep duration at 28 days post-intervention, mean scores on a standardized measure of parents' and infants' quality of life, changes in stool consistency, number of episodes of infant colic per day, parental perception of colic severity (VAS 0–10), parental perception of quality of life (VAS0-10).

### Statistical analysis

Sample size determination was guided by the primary objective of the study and was based on the approach described in Sung et al. [12], using data subsequently published in Sung et al. [13]. Specifically, we considered as a comparison base, and as a proxy for a physiological reduction in crying, the success rate (defined as a reduction in crying time by at least 50%) in the placebo group, which Sung et al. [13] reported to be 39%. In comparison, the success rate observed in subjects treated with probiotics was 66%. Therefore, a sample size of 37 subjects was found sufficient to achieve 90% power to detect a success rate of 0.66 (assuming under the null hypothesis a proportion of 0.39), using a two-sided exact test with a 5% significance level. Considering a dropout rate of 10%, 41 patients were enrolled.

Quantitative variables were summarized using mean ± standard deviation or median and interquartile range, depending on the variable distribution. Qualitative variables were presented using absolute frequencies and percentages.

To compare the duration of crying and various scores over time, Friedman's test was used. Additionally, mixed-effects models were employed to evaluate potential interactions between time and grouping variables.

Statistical analysis was performed using the R statistical software. All statistical tests were conducted at a 5% significance level.

## Results

From July to October 2023, 41 infants were enrolled, two did not complete the study, all children were born full term and median age was 1 months. All baseline characteristics of the enrolled infants are shown in Table 1.

**Table 1:** Baseline characteristics of the enrolled

	**Overall (*N* = 39)**
Age—Median (Q_1_, Q_3_)	1.0 (1.0, 2.0)
Type of Delivery – n (%)	
Natural childbirth	17 (43.6%)
Cesarean child birth	22 (56.4%)
Apgar—Median (Q_1_, Q_3_)	9.0 (8.0, 9.0)
Gestational Age—Median (Q_1_, Q_3_)	39.0 (38.0, 40.0)
Birth Weight—Mean (SD)	3.2 (0.5)
Length At Birth—Mean (SD)	49.7 (1.8)

As primary outcome, we observed in our sample a success rate of 89%, which is significantly higher (*p* < 0.001) compared to the physiological reduction in newborn/infant crying, estimated at 39% [10]. Mean daily crying time at visit 1 was 55 min/day (36.2, 87.5) while at the end of the treatment we observed a reduction to 5 min/day (0, 10) (*p* < 0.001) (Table 2).

**Table 2:** Crying/fussing time, diurnal and nocturnal sleep at week1,2,3 and 4

	**Week1 (*n* = 39)**	**Week2 (*n* = 39)**	**Week3 (*n* = 39)**	**Week4 (*n* = 39)**	***P* value**
Crying/Fussing (Min./day)					
Median (Q_1_, Q_3_)	55(36.2,87.5)	30.0 (20, 40)	15 (10, 25)	5 (0, 10)	< 0.001
Crying/Fussing (Ep./Day)					
Median (Q_1_, Q_3_)	5.0 (4.2, 8.0)	4.0 (3.0, 5.0)	2.0 (2.0, 3.0)	1.0 (0.0, 2.0)	< 0.001
Diurnal Sleep (Min./day)					
Median (Q_1_, Q_3_)	495 (390, 537)	540 (445, 580)	580 (445, 600)	600 (380, 600)	< 0.001
Nocturnal Sleep (Min./day)					
Median (Q_1_, Q_3_)	360 (330, 412)	410 (340, 506)	420 (360, 540)	480 (420, 600)	< 0.001

**Fig. 1:**
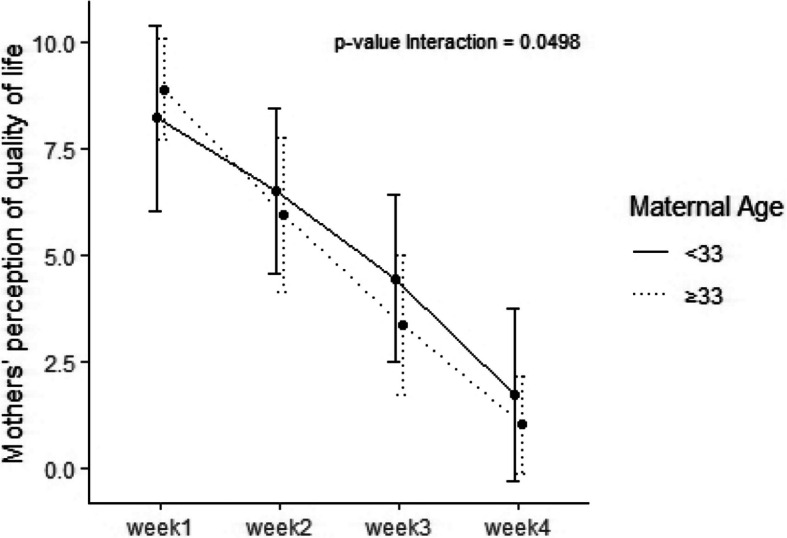
Improvement of quality of life over time in mothers less then 33 years compared to mothers > 33 years

**Fig. 2:**
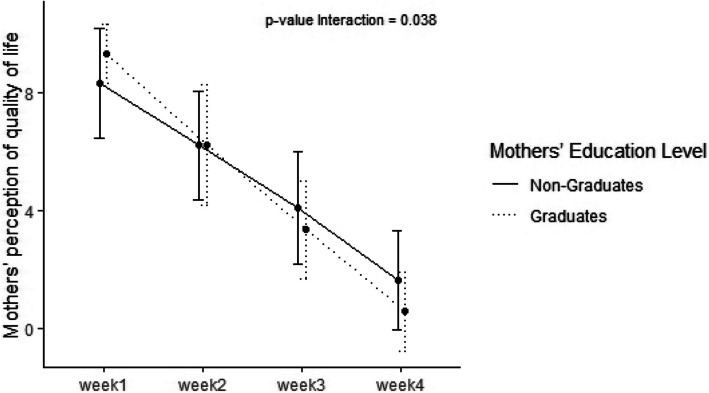
Improvement of quality of life over time in mothers with a degree compared to mothers without a degree

Regarding sleep duration we found that diurnal sleep improved from 495 min/day (390, 537) at visit 1 to 600 min/day (380, 600) (*p* < 0.001) and nocturnal improved from 360 min/day (330, 412) to 480 min/day (420, 600) *p* < 0.001, respectively) (Table 2). In addition, a significant improvement of mothers' and fathers’ perception of quality of life was detected at 28th day (Table 3). We found that the improvement of quality of life over time was significantly larger in mothers with a degree compared to mothers without a degree (*p* = 0.0498) and in mothers aged over 33 years compared to mothers aged under 33 (*p* = 0.038) (Figs. 1 and 2). Furthermore, mothers’ and fathers’ perception of colic severity improved at the end of the study (Table 4).

**Table 3:** Mothers' and fathers’ perception of quality of life

	**Week 1**	**Week 2**	**Week 3**	**Week 4**	***p* value**
Mothers’ perception of quality of life					< 0.001
Median (Q_1_, Q_3_)	8.5 (8.0, 10.0)	6.0 (5.0, 8.0)	4.0 (2.2, 5.0)	1.0 (0.0, 2.0)	
Fathers’ perception of quality of life					< 0.001
Median (Q_1_, Q_3_)	8.0 (8.0, 10.0)	6.0 (5.0, 8.0)	4.0 (3.0, 5.0)	1.0 (0.0, 2.0)

**Table 4:** Mothers’ and fathers’ perceptions of IC severity

	**Week 1**	**Week 2**	**Week 3**	**Week 4**	***p* value**
Mothers’perception of IC severity					< 0.001
Median (Q_1_, Q_3_)	9.5 (8.0, 10.0)	7.0 (6.0, 8.0)	4.0 (3.0, 5.0)	1.0 (0.0, 2.0)	
Fathers’ perception of IC severity					< 0.001
Median (Q_1_, Q_3_)	8.5 (8.0, 10.0)	6.0 (5.0, 8.0)	4.0 (3.0, 5.0)	1.0 (0.0, 2.0)	

With regards to the stool consistency (watery, soft, formed, hard) we found significantly formed stool at 28th day of intervention (Table 5). No relevant adverse event were observed.

**Table 5:** Stool consistency

	**Week 1**	**Week 2**	**Week 3**	**Week 4**	***p* value**
Stool Consistency					
Median (Q_1_, Q_3_)	3.0 (2.0, 4.0)	3.0 (2.0, 3.0)	2.0 (2.0, 3.0)	2.0 (2.0, 2.0)	< 0.001

## Discussion

To our knowledge this is the first study which demonstrates the efficacy and safety of a mixture of Symethicone and Tyndallized Bacillus Coagulans in IC. The result of the present study indicates that the administration of this product is effective in the treatment of infant colic. More specifically we recorded a clinical and statistical significant reduction of infant crying time at 28 days. In a recent survey, van Tilburg et al. reported that the prevalence of IC in children < 1 year of age was 5.9% [17]. Conversely, in another review, some experts in the field hypothesized a likely prevalence of 20%, on the basis of existing literature [18]. Despite the high prevalence and the elevated costs for national healthcare systems, the self-limiting nature of colic has limited the investigations to establish a pathophysiologic model for IC. However, IC has a stressful effect on both infants and their parents, the risk of shaken baby syndrome, self-medication and inappropriate hospitalizations is very high. For all these reasons, it is important to find products that are free of side effects but effective in reducing the symptoms of colic, the duration of crying and increasing sleep time both during the day and at night.

In our study, we observed that the administration of this mixture of Symethicone and Tyndallized Bacillus Coagulans allows an improvement in daytime and nighttime sleep, too. The hours of sleep during the day and the night statistically increased after 28 days of treatment. Parents’ concerns about their child’s sleep are highest in the first year of life [19], with approximately a third of the parents rating their infants’ sleep as problematic [20, 21]. An understanding of parents’ perception of infant sleep problems is critical as they can negatively impact parents, being associated with decreased emotional stability, fatigue and marital difficulty [22]. The most common reported concerns are frequent night waking, difficulty settling to sleep and total night sleep duration [21]. The perception of problematic infant sleep is shaped by the parents’ own confidence with managing infant sleep [22], antenatal and postnatal maternal mental health and maternal sleep quality and daytime functioning [23]. For these reasons, our results seem very important in the management of colicky infants.

Over the decades simethicone has been frequently used for the management of colic. As a matter of fact, data on the effect of the various pharmacological agents are scarce, and evidence for the use of one of those agents in infant colic is weak owing to bias and methodological limitations. Therefore, treatment with pharmacological agents is not supported [24]. The medical device we tested was a mixture of Simethicone and Tyndallized Bacillus Coagulans, the beneficial effects of probiotic supplementation in addition to simethicone in this study may be related to the action of intestinal lactobacilli on the altered balance in infants with colic [25]. Recent studies have shown that modulation of microflora with probiotics, including Bacillus Coagulans, might shift the intestinal ecological balance from potentially harmful flora to flora that would be predominantly beneficial to the host, reducing the risk of gastrointestinal infections and allergic diseases [26]. In particular, probiotic supplementation at an early age aims to provide a safe yet sufficient microbial stimulus for the immature immune system and B.Coagulans has been administered to newborn infants in the attempt to strengthen positive effects associated with colonization by lactobacilli [27].

Concerning well-being and quality of life, we observed a significant improvement of parent’s quality of life and of parent's perception of colic severity at the end of treatment. IC has been frequently associated with higher maternal depression symptom scores and lower quality of life scores [28]. It is also known that the families with a colic infant have less energy and flexibility and more anxiety and problems dealing with daily activities [29, 30]. Moreover, in our study we observed that the improvement of quality of life was significantly higher in mothers with a degree and in mothers aged over 33 years. In our knowledge, this is the first time a positive relationship is done between maternal age and grade of instruction and improvement of maternal quality of life after a treatment for infant colic, which also needs to be re-estalished in a well designed controlled trial.

Strength of this study is a well written protocol. However, our study is not without limitations: this is a pilot study, conducted on a small number of patients. Do to the lack of a control arm, we cannot exclude the possibility of a maturation effect in the crying time reduction, as commonly seen in disorders with a “resolution with time effect”, such as IC. There was also the possibility of a parental bias in reporting modality in our study.

## Conclusions

In summary, infant colic is a common and distressing problem during infancy with effects on the infant, the parents and health-care professionals. When there are no alarm signs or red flags, infant colic should first be treated by giving reassurance and explanations to the parents. However, it could happen that reassurance is not enough, so it is important, in our opinion, to have some safe and effective medical device to suggest. However, this is just a pilot study conducted on a few numbers of patients, so further and larger trials are required. Because this study showed promising results, it would be worthwhile to perform a randomized control trial to unravel the efficacy of this mixture of simethicone and L. coagulans in children with IC.

## Data Availability

The datasets generated during and/or analysed during the current study are not publicly available due [REASON WHY DATA ARE NOT PUBLIC] but are available from the corresponding author on reasonable request.
